# Endolymphatic sac tumor: case report and literature review

**DOI:** 10.1186/s41016-020-00191-4

**Published:** 2020-05-09

**Authors:** Hongliang Ge, Hongyao Wang, Jiawei Cai, Xinting Zhang, Wenzhong Mei, Xiyue Wu, Dezhi Kang

**Affiliations:** 1 Department of Neurosurgery, The First Affiliated Hospital of Fujian Medical University, Fuzhou, 350005 Fujian Province China; 2 Department of Pathology, The First Affiliated Hospital of Fujian Medical University, Fuzhou, 350005 Fujian Province China

**Keywords:** Endolymphatic sac tumor, ELST, Von Hippel-Lindau disease, Temporal bone, Posterior fossae

## Abstract

**Background:**

Endolymphatic sac tumor (ELST) is one of neuroectodermal tumor which arising from endolymphatic sac and duct. It is actually quite rare, with less than 200 cases reported. Although ELST presents benign appearance in histopathology, it can present aggressive destructive behavior in clinical. The cornerstone of treatment for ELST is complete surgical excision. However, it is almost impossible to completely resect the advanced stage tumor. There is still controversy about other treatments, such as radiotherapy and gamma knife surgery.

**Case presentation:**

A 47-year-old man was admitted in The First Affiliated Hospital of Fujian Medical University with a 7-year history of progressive hearing loss and near 6-month repeated attacks of headache. Preoperative CT revealed a massive intracranial lesion and associated hydrocephalus. MR scanning demonstrated a 7.2 cm × 4.6 cm × 4.2 cm bulky mass located in left-sided posterior cranial fossa and temporo-occipital region which showed hyperintensity on T1-weighted images and mixed signal intensity on T2-weighted images. There was no neither clinical manifestation nor family history of Von Hippel–Lindau syndrome (VHL).Due to the mass that was large and invading the bone of skull base, it was difficult to extirpate surgically, so the ventriculoperitoneal shunt combined with local biopsy was performed. The postoperative pathology and immunohistochemical findings confirmed the lesion was an endolymphatic sac tumor. After operation, the patient regularly received radiotherapy.

**Conclusion:**

The widely accepted management of ELST is complete surgical resection. However, it is difficult for surgeons to achieve radical resection with late-stage ELST. Currently, there is much dispute about the role of radiotherapy for the management of ELST in academic circles. In this case where the mass cannot be surgical removed, radiotherapy has the curative effect for ELST in terms of disease control and quality of life.

## Background

Endolymphatic sac tumor (ELST) is an exceedingly rare tumor of the epithelium of endolymphatic ducts and sacs (EDS) with less than 200 cases reported. Although histologically benign, ELST presents clinically invasion behavior because of the disease’s potential for locally infiltration and destruction of bone. It can be occurred in isolation or rather, in simultaneity with Von Hippel–Lindau syndrome (VHL) [1]. The incidence rate of ELST in VHL patients is approximately 10 percent. Consequently, it is valuable to routinely screen for this lesion in VHL patients. The clinical presentation of ELST is mainly characterized by progressive hearing loss with or without cranial neuropathies and depends on the location and extent of brain tissue involvement. It has been accepted that complete surgical excision is the mainstay of management for ELST, whereas it is difficult to drastically remove the late-stage lesion. Controversy over the effect of radiotherapy and gamma knife has been remained. In this report, we describe radiotherapy achieved the desired therapeutic effect for a bulky, unresectable advanced ELST.

## Case presentation

The patient was a 47-year-old man, from The First Affiliated Hospital of Fujian Medical University, Department of Neurosurgery with the chief complaint of progressive left hearing impairment for almost 7 years. Over the past 6 months, the symptom was aggravating that accompanied with repeated occipital headaches. On physical examination, the left ear entirely suffer hearing loss. The hearing of the right ear was normal. The patient presented facial paralysis (House-Brackmann grade II) and hypoglossus paralysis. There was no tinnitus, otalgia, otorrhea, vertigo, and history of trauma or surgeries. In addition, the patient had no definite symptoms or family history of VHL disease.

**Fig. 1:**
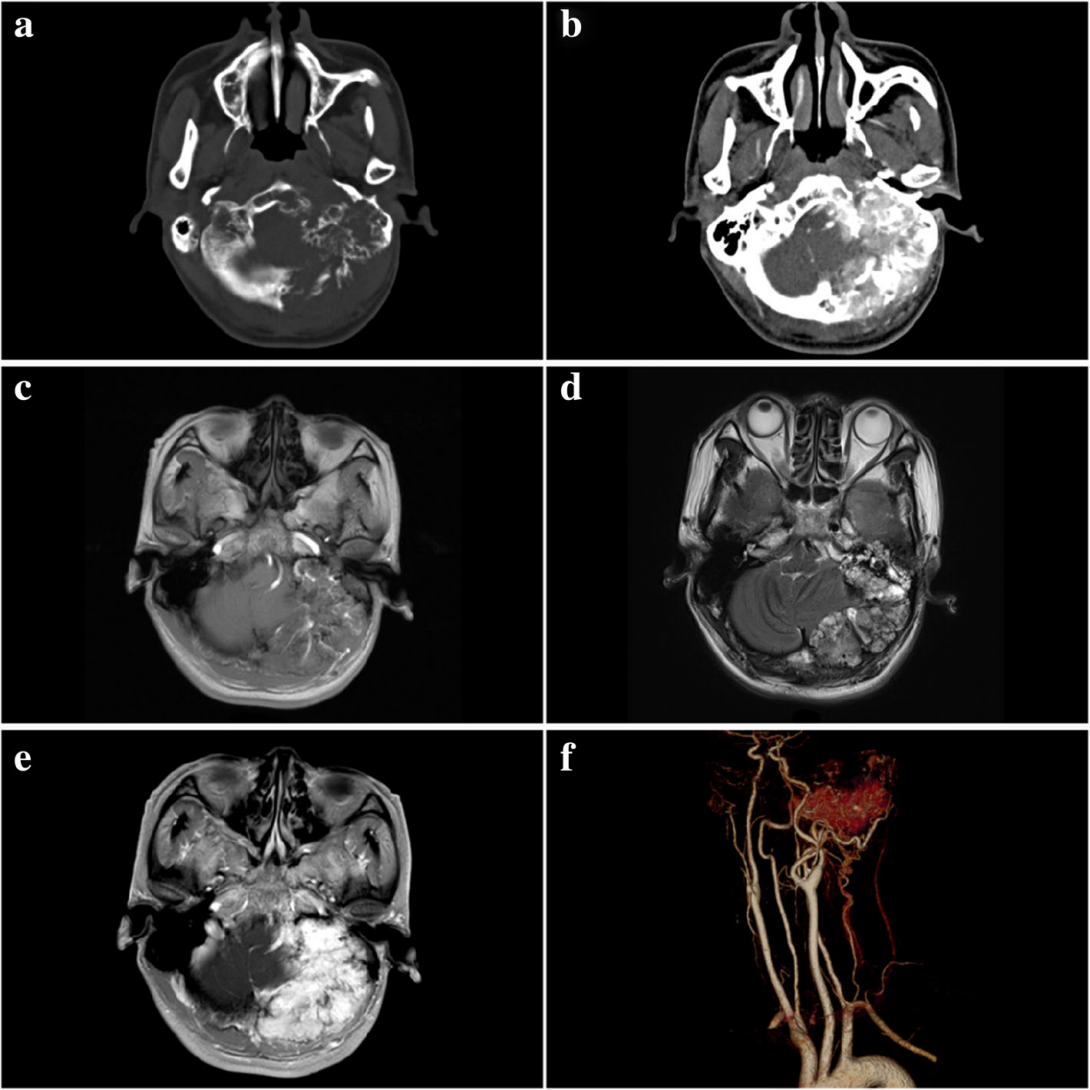
Radiologic characterization of endolymphatic sac tumor. **a**, **b** CT in the axial plane demonstrated an expansile lytic lesion of left mastoid process and middle ear extending posteriorly to the posterior cranial fossa (**a**) with apparent enhancement in contrast imaging (**b**). **c**–**e** MR in the axial plane showed the lesion to be uniformity hypo-signal on the T1-weighted (**c**) and high-low mixed signs on the T2-weighted (**d**), and obviously enhanced with contrast (**e**). **f** Three-dimensional computerized tomographic angiography ( 3D-CTA ) manifested main blood supply of lesion was from the external carotid artery and branches of the subclavian artery

Cerebral computed tomography (CT) findings revealed a bulky enhancing soft tissue mass (maximum transverse diameter was 7.2 cm × 4.6 cm) of left-sided posterior cranial fossa and temporo-occipital region tha t had eroded the skull base bone of middle and posterior cranial fossa (Fig. 1a, b). Linear spiculated density within the lesion represents calcification and residual bone after destructive invasion. Meanwhile, the lesion had compressed the fourth ventricle resulted in hydrocephalus. Cerebral and cervical CT angiography (CTA) revealed the lesion was hypervascular, which main blood supply was from external carotid artery and branches of the subclavian artery, and shadowed of draining veins were visible around the mass, simultaneously (Fig. 1f). Magnetic resonance imaging (MRI) revealed a patchy, massive lesion with irregular, heterogeneous, and lobulated in the left posterior cranial fossa and temporo-occipital region which was showed hypointensity on T1 images (Fig. 1c) and mixed signal intensity on T2-weighted images (Fig. 1d). Magnetic resonance diffusion-weighted imaging (DWI) presented hypointense signals. When given contrast, the lesion obviously showed heterogenous enhancement (Fig. 1e).

**Fig. 2:**
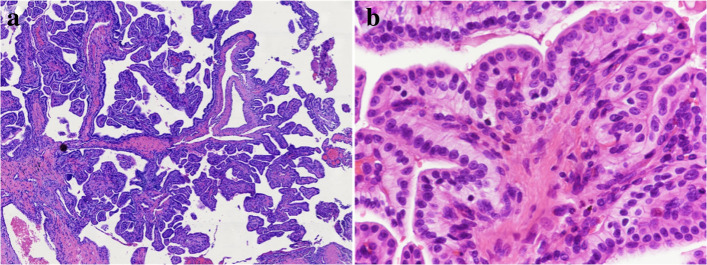
Histopathologic examination of ELST. **a** Histologic sections revealed a papillary architecture and scattered follicular structures (Hematoxylin-eosin, × 100). **b** The papillary structures were lined by a single layer offlattenedcuboidal-to-columnar cells. There were mild cellular pleomorphism and rare mitotic activity (Hematoxylin-eosin, × 400)

**Fig. 3:**
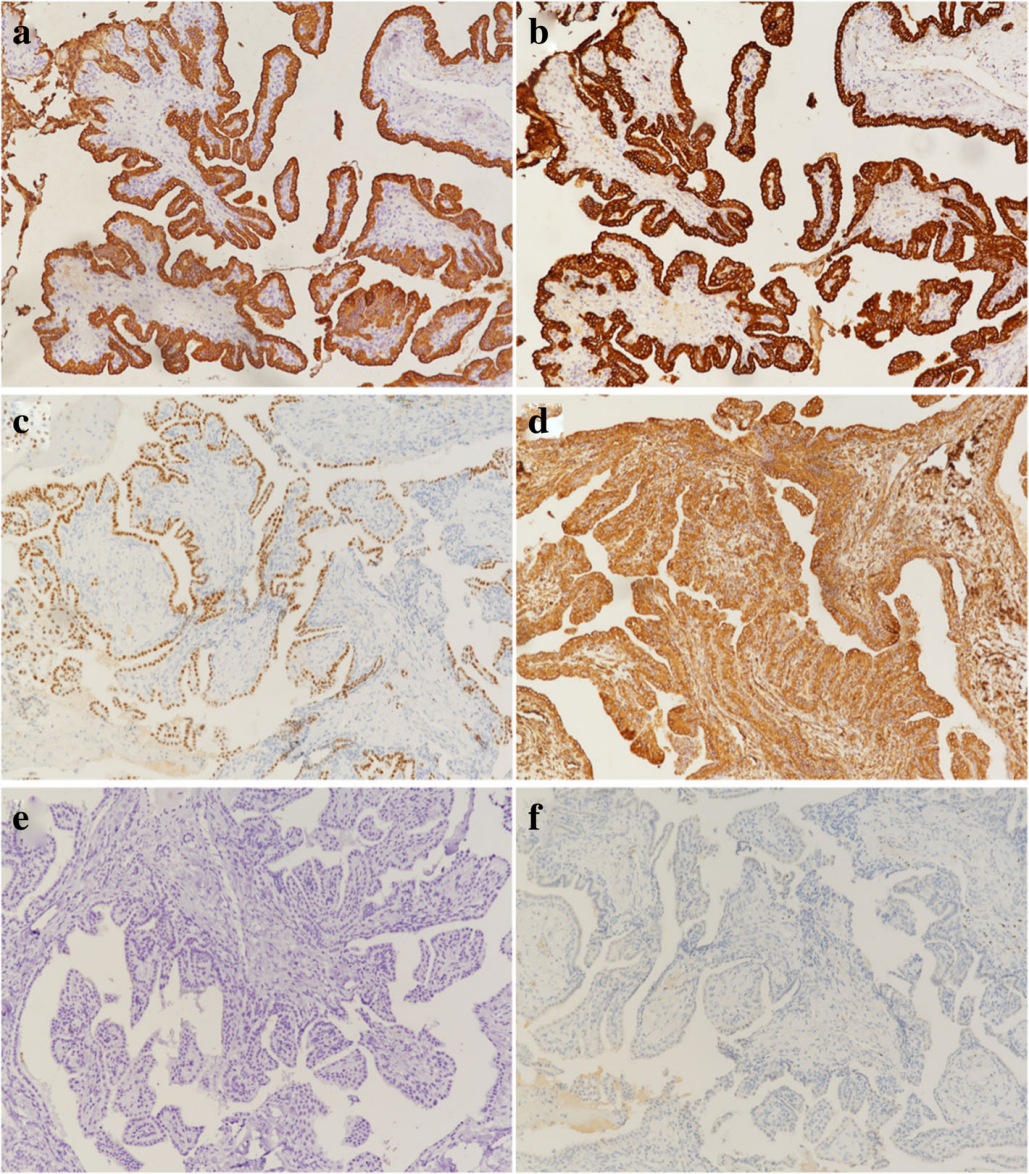
Immunohistochemical staining of ELST. The tumor cells showed positive reactivity with cytokeratin (Pan) (**a**), cytokeratin 7 (**b**), SOX10 (**c**), and vimentin (**d**). Positive reactivity of thyroglobulin (**e**) and S-100 (**f**) was not observed

Because of the tumor that was too large which invading nearby structures, it was difficult to excise completely. Finally, the ventriculoperitoneal shunt combined with local biopsy was performed to cure the hydrocephalus. Postoperative pathology analysis revealed that the tumor cells mainly showed papillary arrangements (Fig. 2a). At high power microscopic view, the papillary structures were found lined by simple cuboidal to columnar epithelioid cells (Fig. 2b). The tumor showed minimal nuclear atypia, rare mitotic activity, and no obvious appearance of necrosis. The results of immunohistochemical examination revealed diffusely positive reactivity with cytokeratin (Pan) (Fig. 3a), cytokeratin 7 (Fig. 3b), SOX10 (Fig. 3c), vimentin (Fig. 3d), 5-lipoxygenase, Oligo-2, and also showed tiny positive reactivity with GFAP. Ki-67 was positive in about 2%, and p53 was positive in 80% on the tumor cells. The immunoreactivity of thyroglobulin (Fig. 3e) and S-100 (Fig. 3f) was negative. As the results of morphologic and immunohistochemical features, the neoplasm was confirmed with pathological diagnosis of endolymphatic sac tumor.

There was no neurologic deficit in the early stage after operation, and the patient got out of the hospital at 7 days postoperatively. Adjuvant radiotherapy was given for palliative treatment postoperatively. In his postoperative 12-month follow-up, interval re-examination MRI scan revealed that the mass remained progression-free and was not leaded to any problem.

## Discussion

Endolymphatic sac tumor is a rare form of benign tumor arising from endolymphatic duct and sac (EDS) [2]. It has previously been referred to as papillary adenomatous tumor, middle ear adenocarcinomas, papillary tumor of temporal bone, adenocarcinoma of endolymphatic sac, and aggressive papillary middle ear tumor, but these names cannot serve as an accurate classification for current understanding of ELST.

ELST can be occurred in isolation or rather, in simultaneity with Von Hippel–Lindau (VHL) disease. VHL disease was named for the German ophthalmologist Eugene Von Hippel and the Swedish pathologist Avrid Lindau, who respectively described the disorders in 1904 and 1927, but it was not widely recognized until 1970s. VHL disease is an autosomal dominant genetic disorder characterized by cysts and benign tumors of multiple systems with potential for subsequent malignant transformation. By researching the genomics and proteomics, the mechanism of VHL disease has been identified. The development of VHL disease has been attributed to mutations of the Von Hippel–Lindau tumor suppressor (VHL) gene which encodes a tumor suppressor, VHL protein (pVHL), located at the short arm of chromosome 3 (3p25-p26) [3]. This leads to be the absence of pVHL, which results in the deregulation of hypoxia-induced factor-alpha, and then induces the development of multi-system neoplasm with potential for malignant transformation. The ELST patients represent about 11–30% in VHL patients [4]. Therefore, VHL patients are commonly indispensable to screen for this disease. In 2004, a retrospective study on ELST by Bambakidis et al. proposed an anatomic classification system which was categorized into four grades in the light of extent and location of tumor. In this previous study, tumor grades in these ELST patients with accompanied VHL disease were lower than that of patients without VHL disease obviously, and mean age was simultaneously lower in VHL patients (31.3 years) than in non-VHL patients (52.5 years) [5]. Although the female-to-male ratio of ELST is considered to be approximately equivalent, some scholars suggest a slight female predominance in VHL disease patients [6, 7].

The ubiquitous clinical symptoms of ELST are one-sided sensorineural hearing loss (SNHL) which may mimick Meniere’s disease, and its mechanism attributes to progressive of tumor associated with either the pressure of adjoining auditory structures or endolymphatic hydrops. It is a gradual process, although acute onset has also been reported. Patients may also present tinnitus, vertigo, aural fullness, and cranial neuropathies, which consist of cranial nerve paralysis, jugular foramen syndrome (glossopharyngeal neuralgia, paralysis of the XII nerve, motor deficit related to the X nerve), and cerebellopontine angle syndrome (hearing loss, facial paralysis, and dizziness) [3, 4, 8].

Representative radiographic findings of ELST are displayed as destruction of bone and markedly inhomogeneous enhancement on CT scan. The neoplasm is situated in the region between posteromedial petrous bone and the sigmoid sinus, which intratumor and posterior rim calcification can be often seen. On MRI, ELST reveals heterogenous signal intensity on T1WI and T2WI. The hemorrhagic areas with sediment of methemoglobin and hemosiderin are markedly hyperintense on T1WI and T2WI. The hypointense areas may attribute to local necrosis, calcifications, or residual bone. In enhanced scan, ELST shows a heterogeneous intensity on T1WI and displays flow voids on T2WI [9, 10]. Angiographic evaluation is not only helpful to conduce to the diagnosis of ELST, but also provide important guidance for interventional embolism [3]. One of the characteristic of ELST is that it is hypervascular which the main supplying arteries arise from external carotid artery system involving branches of the ascending pharyngeal artery or occipital artery [11]. In sporadic cases, there are reported that the blood supply for ELST is in the internal carotid artery system. Preoperative embolization contributes to reduce intraoperative bleeding volume. Even with these clinical and radiological features, the diagnosis of ELST is difficult to distinguish from other lesions, and histopathological examination is the definite method to make diagnosis.

The major characteristics of pathology of ELST are low-grade, locally destructive, richly vascularized, ill-defined margins. The gross of ELST representatively appears as soft, reddish-blue polypoid lesion; microscopically, ELST shows two fundamental forms: papillary and follicular. The papillary pattern is arranged by simple cuboidal or columnar neoplastic epithelium cells. By comparison, the follicular pattern exhibits cystic filled with colloid protein material. Some cases had been reported that ELST may present both patterns [12, 13]. Cytological observations showed that there is rare mitosis and no areas of necrosis.

The histological differential diagnoses of ELST include middle ear carcinoma, choroid plexus papilloma, paraganglioma, papillary ependymoma, metastatic renal cell carcinoma, and metastatic thyroid papillary carcinoma. The foremost of those is the middle ear carcinoma which expands within mesotympanum, without causing bone invasion and destruction. However, a middle ear carcinoma is different than an ELST in that its cells show distinct pleomorphism and more conspicuous mitotic activity. The differences between ELST and the remaining entities previously described can be distinguished by immunohistochemical staining. ELST is considered to originate from intratemporal portion of the endolymphatic sac which is of neuroectodermal embryogenesis [5, 14]; therefore, immunohistochemical staining of ELST shows positive for cytokeratin and vimentin, and the majority will be S-100 positive. By profiling chromogranin negative immunohistochemistry of ELST, the results will help distinguish ELST from paraganglioma. Transthyretin immunohistochemistry may be aided by differential diagnosis from ELST and choroid plexus tumor. Transthyretin staining in choroid plexus tumor is distinctly positive, whereas ELST exhibits negative detection. Moreover, the negative staining result of thyroglobulin in the lesions is helpful in differentiating ELST from metastatic thyroid cancer [5, 8, 15].

Surgery is still recognized as the cornerstone of treatment which there was no ever reported recurrences after complete surgical resection, whereas subtotal resection may result in local recurrence. But, entire resection of a late-stage ELST is often not possible due to the anatomic complexity and distinct patterns of extension. The common choices of the operative approach include the transmastoid, posterior fossa, or retrolabyrinthine-transdural approaches [16, 17]. The curative effect of radiation and gamma knife surgery remains controversial [18]. Recommendations from some literatures and postoperative radiotherapy may be effective as an adjuvant treatment for cases of recurrent or difficult resection [14, 19].

It has been recommended that at least 10 years of follow-up investigations of ELST patients should consist of annual MRI scanning. However, there is no consensus as to what follow-up should be arranged for ELST patients, although genetic evaluation is deemed necessary to evaluate the future development. There is no treatment protocol for the patients of unresectable or subtotal resections, into which research should be urgently needed, especially late-stage ELST.

## Conclusion

We present this rare case to aim at the better understanding and awareness of this extremely rare neoplasm. Although surgery excision, as standard treatment has been widely accepted, there has been controversial in unresectable cases. Due to the lack of evidence in the literature concerning the role of radiotherapy of unresectable ELST, we would like to put forward that radiotherapy could also control condition progressing; however, there is need to be long follow-up to assess the long term effect of radiotherapy. A multidisciplinary treatment approach including neurosurgeons, radiologists, neuropathologists, and radiation oncologists should be provided to patients with ELST to achieve adequate control of these locally aggressive tumors.

## Data Availability

Not applicable.

## Abbreviations

ELST: Endolymphatic sac tumor
MRI: Magnetic resonance imaging
CT: Computed tomography
VHL: Von Hippel–Lindau syndrome
pVHL: VHL protein
EDS: Endolymphatic duct and sac

## References

[1] Kim HJ, Hagan M, Butman JA, Baggenstos M, Brewer C, Zalewski C (2013). Surgical resection of endolymphatic sac tumors in von Hippel-Lindau disease: findings, results, and indications. Laryngoscope..

[2] Michaels L (2007). Origin of endolymphatic sac tumor. Head Neck Pathol..

[3] Wick CC, Manzoor NF, Semaan MT, Megerian CA (2015). Endolymphatic sac tumors. Otolaryngol Clin North Am..

[4] Yilmaz I, Bolat F, Demirhan B, Aydin V, Ozluoglu LN (2008). Endolymphatic sac papillary tumor: a case report and review. Auris Nasus Larynx..

[5] Bambakidis NC, Megerian CA, Ratcheson RA (2004). Differential grading of endolymphatic sac tumor extension by virtue of von Hippel-Lindau disease status. Otol Neurotol..

[6] Reijneveld J, Hanlo P, Groenewoud G, Jansen G, van Overbeeke K, Tulleken C (1997). Endolymphatic sac tumor: a case report and review of the literature. Surg Neurol..

[7] Hamazaki S, Yoshida M, Yao M, Nagashima Y, Taguchi K, Nakashima H (2001). Mutation of von Hippel-Lindau tumor suppressor gene in a sporadic endolymphatic sac tumor. Hum Pathol..

[8] Devaney KO, Ferlito A, Rinaldo A (2003). Endolymphatic sac tumor (low-grade papillary adenocarcinoma) of the temporal bone. Acta Otolaryngol..

[9] Patel NP, Wiggins RH, Shelton C (2006). The radiologic diagnosis of endolymphatic sac tumors. Laryngoscope..

[10] Mukherji SK, Castillo M (1996). Adenocarcinoma of the endolymphatic sac: imaging features and preoperative embolization. Neuroradiology..

[11] Joy HM, Barker CS, Millar JS, Davis A (2002). Radiological considerations in the diagnosis of an endolymphatic sac tumour. Clin Radiol..

[12] Lonser RR, Kim HJ, Butman JA, Vortmeyer AO, Choo DI, Oldfield EH (2004). Tumors of the endolymphatic sac in von Hippel-Lindau disease. N Engl J Med..

[13] Megerian CA, McKenna MJ, Nuss RC, Maniglia AJ, Ojemann RG, Pilch BZ (1995). Endolymphatic sac tumors: histopathologic confirmation, clinical characterization, and implication in von Hippel-Lindau disease. Laryngoscope..

[14] Kupferman ME, Bigelow DC, Carpentieri DF, Bilaniuk LT, Kazahaya K (2004). Endolymphatic sac tumor in a 4-year-old boy. Otol Neurotol..

[15] Megerian CA, Pilch BZ, Bhan AK, McKenna MJ (1997). Differential expression of transthyretin in papillary tumors of the endolymphatic sac and choroid plexus. Laryngoscope..

[16] Kim HJ, Butman JA, Brewer C, Zalewski C, Vortmeyer AO, Glenn G (2005). Tumors of the endolymphatic sac in patients with von Hippel-Lindau disease: implications for their natural history, diagnosis, and treatment. J Neurosurg..

[17] Megerian CA, Haynes DS, Poe DS, Choo DI, Keriakas TJ, Glasscock ME (2002). Hearing preservation surgery for small endolymphatic sac tumors in patients with von Hippel-Lindau syndrome. Otol Neurotol..

[18] Cheng X, Qin S, Wang X, Li P, Shrestha B, Wang W (2011). Gamma knife treatment of an endolymphatic sac tumor: unique features of a case and review of the literature. Neurol India..

[19] Folker RJ, Meyerhoff WL, Rushing EJ (1997). Aggressive papillary adenoma of the cerebellopontine angle: case report of an endolymphatic sac tumor. Am J Otolaryngol..

